# The cost burden of trastuzumab and bevacizumab therapy for solid tumours in Canada

**DOI:** 10.3747/co.v15i3.249

**Published:** 2008-06

**Authors:** A. Drucker, C. Skedgel, K. Virik, D. Rayson, M. Sellon, T. Younis

**Affiliations:** * Department of Medicine, Dalhousie University, Halifax, NS; † Division of Medical Oncology, QEII Health Sciences Centre, Halifax, NS; ‡ Department of Pharmacy, QEII Health Sciences Centre, Halifax, NS

**Keywords:** Health care costs, monoclonal antibodies, trastuzumab, bevacizumab, neoplasms

## Abstract

**Objective:**

Monoclonal antibodies (MAbs) such as trastuzumab and bevacizumab have become important yet expensive components of systemic cancer therapy across a variety of disease sites. We assessed the potential cost implications of adopting trastuzumab and bevacizumab therapy in the context of their potential utilization in breast, lung, and colorectal cancers.

**Design:**

We first estimated MAb costs per patient and treatment indication and then included the MAb acquisition cost and the costs of medical resource utilizations required for therapy delivery. Drug costs were based on 2005 average Canadian wholesale prices, assuming full drug delivery and uncomplicated cycles. A direct-payer perspective was undertaken, and results are reported in Canadian dollars. Potential lifetime costs were then derived according to constructed schema, which account for absolute numbers of target patients and systemic therapy utilization. We subsequently estimated costs of MAb therapy relative to total costs of conventional management without MAb therapy.

**Results:**

Trastuzumab costs $49,915 and $28,350 per patient treated in the adjuvant and metastatic breast cancer settings, respectively; bevacizumab costs $48,490 and $39,614 per patient treated in the metastatic lung and colorectal cancer settings, respectively. Potential lifetime absolute costs to Canada’s health care system were approximately $127 million and $299 million for trastuzumab and bevacizumab respectively, corresponding to an average increase in health care expenditure of approximately 19% for breast cancer and 21% for lung and colorectal cancer over conventional management without MAbs.

**Conclusions:**

Novel Mab-based therapies such as trastuzumab and bevacizumab will likely add a significant cost burden to Canada’s publicly funded health care system.

## 1. INTRODUCTION

Cancer treatment costs have increased dramatically since the late 1990s, escalating particularly since 2000 1, with the increasingly regular introduction of novel and expensive systemic therapies. Cancer prevalence rates and the numbers of cancer patients receiving active therapy are also increasing annually 1,2. In light of these observations, cancer treatment costs are expected to continue to rise exponentially over subsequent decades. These projections have raised concerns regarding the sustainability of cancer care funding, particularly within a single-payer public system such as exists in Canada. Meanwhile, an increased emphasis has been placed on economic evaluations of newer therapies through either cost-effectiveness or budget-impact approaches; such evaluations now play an increasingly important role in funding decision-making for novel systemic therapies.

The introduction of targeted therapeutic agents such as monoclonal antibodies for solid tumours has revolutionized cancer care and is rapidly changing treatment approaches across common cancer disease sites. Trastuzumab and bevacizumab are two such agents that have been shown to improve disease-free, progression-free, or overall survival in breast, colorectal, and lung cancer 3–7. Although both agents were approved by Health Canada, considerable controversy surrounding their cost and cost-effectiveness remains 8–12, leading to regional disparity in access across Canada. For instance, trastuzumab is funded by all Canadian provinces for the adjuvant and palliative treatment of human epidermal growth factor receptor type 2 (her2/*neu*)–positive breast cancer, but bevacizumab is funded in only a few provinces for the palliative treatment of metastatic colorectal cancer. This discrepancy highlights provincial differences in funding and has become an important issue in discussions regarding a national strategy for equitable drug funding in cancer care (Canadian Partnership Against Cancer) proposed by the current federal government 13.

Because of high acquisition costs, trastuzumab and bevacizumab could add significant economic burdens to all provincial health budgets in Canada. However, the estimated cost potentially incurred with their use across cancer-specific disease sites is not currently known. We undertook an economic analysis to estimate the incremental costs potentially associated with the use of trastuzumab and bevacizumab for breast, colorectal, and lung cancers in Canada.

## 2. METHODS

**FIGURE 1:**
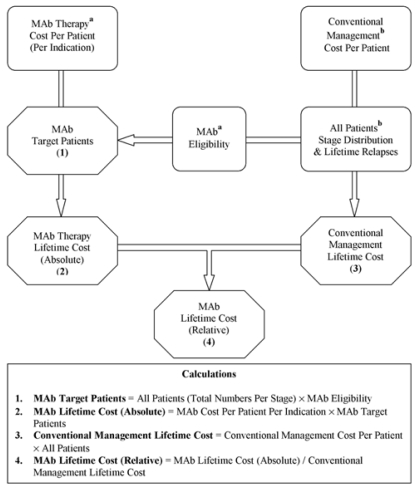
Methods schema. MAb = monoclonal antibody. ^a^ Estimated as described in “Methods” and in Tables.I and IV ^b^ Derived from the literature with adjustments, and as described in Figure 2.

Costs potentially associated with the use of trastuzumab and bevacizumab in breast, colorectal, and non-small-cell lung (nsclc) cancers in Canada were estimated according to the schema outlined in Figure 1 and are described in the subsections that follow. The aim of our study was to assess the additional costs incurred with these monoclonal antibodies according to their estimated utilization rates. Because these novel therapies are used in addition to, and not as a replacement for, existing systemic therapies for their respective malignancies, clinical efficacy data were neither modeled nor required. A direct-payer perspective was undertaken, and results are reported in 2005 Canadian dollars.

### 2.1 Costs Associated with Monoclonal Antibody Therapy per Patient per Indication

The total quantity of monoclonal antibody use, in milligrams, per patient per indication was calculated based on the recommended dose and schedule of treatment and the expected duration of therapy (Table I).A number of assumptions were made, as delineated in Table II.

The estimated total quantity of monoclonal antibody used per patient per indication (that is, total quantity in milligrams) was multiplied by the 2005 average wholesale price in Canada for the relevant monoclonal antibody (that is, unit cost in 2005 Canadian dollars) to calculate the monoclonal antibody cost per patient per indication (Table I).

We also estimated the additional cost of medical resource utilizations required for treatment delivery, including costs associated with necessary supportive medications, diagnostic investigations, and human resources required during clinic and chemotherapy suite visits to administer the monoclonal antibody. Unit costs were derived from local resources at the QEII Health Sciences Centre in Halifax, Canada (Table III). Resource utilization was estimated based on current local practice and on the relevant clinical trial treatment algorithms from which efficacy data were obtained.

The estimated costs of trastuzumab therapy for metastatic breast cancer were validated by reviewing pharmacy utilization records at the QEII Health Sciences Centre for patients with her2/*neu-*positive meta-static breast cancer 15. A similar validation review was not possible for bevacizumab or adjuvant trastuzumab, because the necessary data were not locally available at the time of analysis.

### 2.2 Lifetime Costs Associated with Monoclonal Antibody Therapy in Canada

**FIGURE 2:**
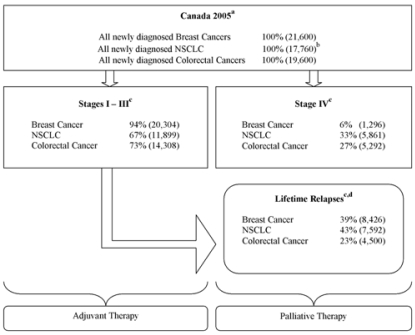
Total numbers and stage distributions for breast, non-small-cell lung (nsclc), and colorectal cancers. ^a^ Canadian Cancer Statistics 2005 1. ^b^ Estimated at 80% of the 22,200 patients diagnosed with all types of lung cancer 26. ^c^ Stage distribution and future relapses were derived from the literature for breast 17, colorectal 19, and nsclc 7 cancers. ^d^ Lifetime relapses represent the number of patients diagnosed with early-stage (i–iii) cancer (nonmetastatic) multiplied by the expected lifetime risk of relapse for these patients. The estimated numbers for nsclc represent patients with local and distant relapses, because these patients were considered potential candidates for bevacizumab. The estimated numbers for breast and colorectal cancers reflect patients with distant relapses only, because these patients were assumed to be eligible for palliative trastuzumab or bevacizumab monoclonal antibody therapy.

Lifetime costs associated with potential trastuzumab and bevacizumab treatment for patients diagnosed with breast, lung, and colorectal cancers over a 1-year period in Canada were estimated. Potential repeat utilizations upon relapse (that is, palliative treatment) after treatment initially given as adjuvant therapy were included. The stage distribution and disease course, including potential relapses, were derived from *Canadian Cancer Statistics* and the literature as described in Figure 2.

Absolute and relative costs were both examined (Figure 1). Absolute lifetime cost was defined as the incremental cost associated with monoclonal antibody therapy for patients over the course of their disease.

Relative lifetime cost was defined as a ratio of this absolute lifetime cost to the absolute lifetime cost for conventional management without monoclonal antibodies. The time horizon of this budget impact analysis was that of a lifetime horizon for the study cohort, ranging from several years in metastatic cancer to decades in patients who are potentially cured with adjuvant treatment. Lifetime costs of conventional management per patient with stages i–iii and iv disease were derived from the literature with adjustments: $29,868 and $42,532 for breast cancer, $26,980 and $28,790 for nsclc, and $39,925 and $65,390 for colorectal cancer respectively 16,17,19,21.

A stepwise approach with multiple calculations was used (Figure 1). The number of patients eligible for each monoclonal antibody was estimated based on

- the number of patients diagnosed with breast cancer, colorectal cancer, and nsclc in 2005 in Canada^1^ (Figure 2);
- the respective stage-specific disease distribution at diagnosis and predicted lifetime relapse rates (Figure 2); and
- the proportion of incident cases potentially eligible for each monoclonal antibody therapy per indication (Table IV) according to disease stage and relapse rates.

As an example, the proportion of breast cancer patients eligible for treatment with adjuvant trastuzumab (Table IV) was derived from the proportion of patients eligible to receive adjuvant chemotherapy 22,27 multiplied by the proportion of breast cancer patients with her2/*neu-*positive disease 31, subtracting the proportion of patients excluded because of contraindications to trastuzumab therapy such as underlying cardiac dysfunction.

#### 2.2.1 Absolute Lifetime Cost of Monoclonal Antibody Therapy in Canada

Absolute lifetime cost of monoclonal antibody therapy in Canada was calculated as the product of the monoclonal antibody cost per patient per indication (Table I) and the total number of patients in Canada eligible for the relevant treatment, including those with relapsed disease (Figure 2).

As an example, the absolute lifetime cost associated with trastuzumab for patients diagnosed with breast cancer in 2005 in Canada included the cost of adjuvant trastuzumab treatment for eligible patients diagnosed with stages i–iii disease, plus the costs of palliative trastuzumab therapy for patients with relapsed disease after adjuvant trastuzumab who remained candidates for re-treatment, plus the costs of palliative trastuzumab therapy for eligible patients diagnosed with metastatic disease at first presentation (Figure 2).

To validate these results, we compared our estimates of the absolute lifetime cost of trastuzumab with total trastuzumab sales in Canada for 2006, as obtained through IMS Health Canada 35.

#### 2.2.2 Relative Lifetime Cost of Monoclonal Antibody Therapy in Canada

Absolute lifetime cost of conventional management (that is, without monoclonal antibody therapy) per patient for the relevant tumour type was estimated according to disease incidence rates, stage distribution, and estimated rates of relapsed disease after potentially curative primary therapy (that is, surgery with or without adjuvant radiation or systemic therapy, or both). Costs were derived from the literature 16–19 and adjusted to 2005 Canadian dollars using the Consumer Price Index health care component 36. A number of adjustments were made to account for conventional systemic therapies newly introduced since the estimates were first reported (Table II). The relative lifetime cost of monoclonal antibody therapy was calculated as the ratio of the lifetime cost of monoclonal antibody therapy to the lifetime cost of conventional management alone.

## 3. RESULTS

The incremental cost of adjuvant and palliative therapy with trastuzumab for her2/*neu-*positive breast cancer was estimated to be $49,915 and $28,350 respectively per patient. The incremental cost of bevacizumab was estimated to be $48,490 and $39,614 for the treatment of metastatic nsclc and metastatic colorectal cancer respectively per patient. These costs reflect primarily the acquisition costs of monoclonal antibodies, which accounted for 94%–97% of estimated total costs (Table I).

Nationally, the absolute lifetime cost associated with trastuzumab for the treatment of her2/*neu-*positive breast cancer patients diagnosed in 1 year was estimated to be $127 million. Similarly, the estimated absolute lifetime costs potentially associated with bevacizumab were $117 million for metastatic nsclc and $182 million for metastatic colorectal cancer, for a total estimated lifetime cost of $299 million. Of the total trastuzumab cost, $86.7 million was attributable to adjuvant treatment for early-stage breast cancer as compared with $39.8 million for palliative treatment of advanced or relapsed disease. Treatment with trastuzumab ($127 million) would increase the overall lifetime cost of breast cancer management by 19%, and treatment with bevacizumab ($299 million) could potentially increase the overall lifetime costs of colorectal cancer and nsclc management by 21% relative to the estimated costs of conventional management without monoclonal antibodies for these cancers.

The estimated total drug acquisition cost for palliative trastuzumab per patient based on the assumptions in the present study ($26,648) was approximately 10% higher than the actual average total drug cost per patient previously derived through local pharmacy records ($23,908) 15. As well, the estimated national budget impact for trastuzumab ($127 million) was within 10% of the $138 million total trastuzumab sales in Canada for 2006 35.

## 4. DISCUSSION

Monoclonal antibody therapy for breast, colorectal, and lung cancer in Canada is expected to have significant budgetary implications for Canada’s publicly funded health care system. We estimate that, for each patient treated with adjuvant and palliative trastuzumab, an additional cost of $49,915 and $28,350 respectively will be incurred. Bevacizumab could also add an additional cost per patient of $48,490 and $39,614 for the treatment of metastatic nsclc and metastatic colorectal cancer respectively. These costs could amount to an additional $426 million in lifetime costs across all patients diagnosed with breast, colorectal, and lung cancers in 1 year, corresponding to an approximate 21% increase in the relative lifetime costs of treatment for these diseases. Of these total estimates, the additional $127 million expected with trastuzumab therapy for her2/*neu-*positive breast cancer likely reflects a current effect, because this agent is currently funded in all Canadian provinces; the $299 million estimate for bevacizumab represents a potential rather than an actual effect, because funding for bevacizumab is currently limited to colorectal cancer and is supported by only a few Canadian provinces.

Although our study corroborates the high costs of trastuzumab and bevacizumab, its limitations include the number of assumptions that had to be made and the derivation of the conventional management costs on which we relied. First, in regard to the assumptions made, we did not conduct sensitivity analyses, because such analyses would have required the definition of arbitrary distributions around the point estimates examined and because the input parameters and the resulting cost estimates had a simple linear relationship in our schema. We attempted, however, to validate our estimated costs for trastuzumab based on these assumptions. The validation revealed our estimates to be within 10% of the actual cost of national trastuzumab sales for 2006 and also within 10% of the observed local cost of incorporating trastuzumab for the palliative treatment of her2/*neu-*positive metastatic breast cancer. Second, the conventional management costs used to estimate relative lifetime costs were derived from older studies and were themselves estimates. Since those estimates were first reported, clinical practice has evolved across the three disease sites examined. We attempted to address that evolution by incorporating the costs of newer interventions whenever possible and by adjusting older costs to current costs through the Consumer Price Index 36.

Given limited health care resources, the results of our economic evaluation reinforce concerns of drug affordability on an individual and a population basis. The implication of rising costs of anticancer drugs within relatively fixed provincial health care budgets is that inequity in access to novel anticancer therapies is beginning to arise in Canada. Currently, newer interventions that are not within an acceptable cost-effectiveness threshold 37 in various jurisdictions are often denied funding in an attempt to maximize health outcome gains within a limited budget. However, in an era of rising health care costs and in the context of a plethora of novel promising targeted therapies for cancer, revision of commonly accepted cost–benefit thresholds may have to occur to potentially incorporate more of these expensive yet effective therapies. Targeted anticancer drugs are redefining the management of common malignancies and will likely be the future of cancer treatment. Therefore, provincial and national programs will be forced to somehow adapt to these increasing economic demands on health care budgets.

**TABLE I:** Monoclonal antibody (MAb) costs per patient per indication

Schema component	Trastuzumab Breast		Bevacizumab	
	Adjuvant	Palliative	nsclc Palliative	Colorectal Palliative
Dose a (mg/kg)	8 (1st dose)→6^5^,^6^	8 (1st dose)→6^b^,^3^,^14^	15^7^	5^4^
Schedule a (every *n* weeks)	3^5^,^6^	3^3^,^14^	3^7^	2^4^
Duration a (months)	12^5^,^6^	7.2^3^,^14^,^15^	6.4^7^	10.6^4^
Total use c (mg)	7700	4340	9450	7700
Cost per milligram d ($CA)	6.14	6.14	5.00	5.00
MAb cost e ($CA)	47,279	26,648	47,251	38,501
MRU ($CA)	2,637	1,702	1,239	1,113
Total costs ($CA)	49,916	28,350	48,490	39,614

a Derived from the relevant clinical trials (see Table II).

b Dose schedule as per local practice.

c Calculated based on the dose, schedule, and duration of therapy.

d Drug acquisition costs were based on 2005 average Canadian wholesale prices.

e Calculated based on total use in milligrams multiplied by cost per milligram.

nsclc = non-small-cell lung cancer; $CA = 2005 Canadian dollars; mru = medical resource utilization.

**TABLE II:** Key assumptions

Treatment with monoclonal antibodies (that is, dose, schedule, and duration of therapy) were based on the following clinical trial treatment algorithms:
Adjuvant trastuzumab: 8 mg/kg loading dose followed by 6 mg/kg maintenance every 3 weeks for 1 year after completion of adjuvant chemotherapy (“sequential approach”) based on hera (Herceptin Adjuvant Trial) 5. Palliative trastuzumab: 8 mg/kg loading dose followed by 6 mg/kg maintenance every 3 weeks, as per local practice, for 7.2 months, based on average time to progression from clinical trials 3,14,15. Palliative bevacizumab in colorectal cancer: 5 mg/kg every 2 weeks in combination with chemotherapy for 10.6 months, based on time to progression from relevant clinical trial 4. Palliative bevacizumab in non-small-cell lung cancer (nsclc): 15 mg/kg every 3 weeks in combination with and following chemotherapy, for 6.4 months, based on time to progression from relevant clinical trial 7. Monoclonal antibody doses were calculated based on complete drug delivery for patients with an average body weight of 70 kg.
Medical resources utilization (mru) was estimated based on relevant clinical trial treatment algorithms and on local practice at the QEII Health Sciences Centre in Nova Scotia, Canada (details of unit costs and mru available upon request).
Costs of potential complications secondary to monoclonal antibody treatments were not considered.
Trastuzumab is contraindicated in 5% of patients with her2/*neu* breast cancer (for cardiac dysfunction, for instance).
Bevacizumab is contraindicated in 5% of patients with nsclc and colorectal cancer (for risk of bleeding, thrombosis, or uncontrolled hypertension, for instance).
Bevacizumab is not indicated in nsclc patients with squamous carcinoma.
The estimated disease-stage distribution 16–19 and lifetime relapse rates derived from the literature have not changed over time.
Patients with relapsed disease and stage iv cancer are eligible for palliative therapy.
Patients with relapsed breast cancer are re-treated with palliative trastuzumab if their disease recurred more than 6 months after completion of adjuvant trastuzumab therapy.
Conventional management costs derived from the literature were adjusted to incorporate the costs of newer therapies, which were introduced since the estimates were first reported, according to reported utilization rates:
Adjuvant chemotherapy for early-stage nsclc 20,21. Palliative chemotherapy for stage iv or relapsed nsclc 22–24. Irinotecan-based palliative chemotherapy in stage iv or relapsed colorectal cancer 18,19,25.

**TABLE III:** Medical resources utilization (mru) costs per patient

MRU	Cost a
Trastuzumab—adjuvant breast cancer	
Supportive medications	1
Diagnostic investigations	1454
Human resources	1183
Total	2637
Trastuzumab—metastatic breast cancer	
Supportive medications	1
Diagnostic investigations	1175
Human resources	527
Total	1702
Bevacizumab—metastatic colorectal cancer	
Supportive medications	1
Diagnostic investigations	96
Human resources	1018
Total	1113
Bevacizumab—metastatic NSCLC	
Supportive medications	1
Diagnostic investigations	715
Human resources	524
Total	1239

a In 2005 Canadian dollars.

nsclc = non-small-cell lung cancer.

**TABLE IV:** Monoclonal antibody (MAb) eligibility

Eligibility factors	Trastuzumab Breast		Bevacizumab	
	Adjuvant	Palliative	nsclc Palliative	Colorectal Palliative
Treated with chemotherapy (%)	40^24^,^27^	70^17^	32^23^,^24^,^28^,^29^	50^19^,^25^,^30^
With treatment indication (%)^a^	23^b^,^31^	23^b^,^31^	60^c^,^32^–^34^	100
Excluded (%) d	5	5	5	5
Total eligible (%)	9	15	18	48

a her2/*neu-*positive breast cancer, all nsclc excluding squamous histology, and all colorectal cancers.

b her2/*neu-*positive breast cancer.

c All nsclc, excluding squamous cell histology.

d Assumption secondary to possible cardiac causes or high bleeding risks (see Table II).

nsclc = non-small-cell lung cancer.
